# The Future of the Bologna Process and the European Higher Education Area: New Perspectives on a Recurring Topic

**DOI:** 10.1007/978-3-030-56316-5_23

**Published:** 2020-07-26

**Authors:** Sjur Bergan, Liviu Matei

**Affiliations:** 1 UNESCO Chair on Science and Innovation Policies, National University of Political Studies and Public Administration, Bucharest, Romania; 2 European Commission, Brussels, Belgium; 3 National University of Political Studies and Public Administration, Bucharest, Romania; 4 Council of Europe, Strasbourg, France; 5 Central European University, Budapest, Hungary

## Abstract

The future of the Bologna Process and the European Higher Education Area (EHEA) have been debated for more than 20 years (Bergan and Deca 2018). From the very start, even as the implementation of this continental-wide project in higher education got underway and in parallel to historical analyses (“looking back” too) that begun slowly to emerge, the future of the EHEA has been a constant preoccupation. It is perhaps in the nature of things that while the future can be close or distant, it never quite arrives, like a textitfata morgana, so that any discussion of “the future” can in principle be endless. Or, it could be that in this case discussions about the future indicate continuing uncertainty about the substance, shape and timeline of a European area for higher education. As we are completing the second decade of the Bologna Process and, if we take a formal approach, the first decade of the EHEA, this debate nevertheless takes on added urgency and includes some new elements. We are encouraged by the fact that few if any voices have been heard advocating an end to the EHEA. We therefore disregard this option here.

## The European Higher Education Area at 21: *Fata Morgana* or Continuing Policy Journey?

The future of the Bologna Process and the European Higher Education Area (EHEA) have been debated for more than 20 years (Bergan and Deca 2018).^1^ From the very start, even as the implementation of this continental-wide project in higher education got underway and in parallel to historical analyses (“looking back” too) that begun slowly to emerge, the future of the EHEA has been a constant preoccupation. It is perhaps in the nature of things that while the future can be close or distant, it never quite arrives, like a textitfata morgana, so that any discussion of “the future” can in principle be endless. Or, it could be that in this case discussions about the future indicate continuing uncertainty about the substance, shape and timeline of a European area for higher education. As we are completing the second decade of the Bologna Process and, if we take a formal approach, the first decade of the EHEA, this debate nevertheless takes on added urgency and includes some new elements. We are encouraged by the fact that few if any voices have been heard advocating an end to the EHEA. We therefore disregard this option here.

When the EHEA was formally established a decade ago (Bologna Process 2010), it could be seen as a transition from a development process to a steady state of affairs. In this view, the EHEA would be seen as an established common area with defined characteristics, such as a European-wide overarching qualifications framework, agreed standards for quality assurance and the recognition of qualifications, a common understanding of the social dimension of higher education, and a clear “foreign policy” defined by the “global dimension” strategy (Bologna Process 2007) and the Bologna Policy Forum,^2^ which was launched in 2009. Not least, the EHEA is based on a set of fundamental values accepted by all its members and expected to be respected by all. In the Paris Communiqué, these are described as follows:

Academic freedom and integrity, institutional autonomy, participation of students and staff in higher education governance, and public responsibility for and of higher education form the backbone of the EHEA (Bologna Process 2018a: 1).

These values are a slight development of what the Bologna Follow Up Group (BFUG^3^) in 2004 described as the “principles underpinning the Bologna Process”:

- Mobility of students and staff;
- Autonomous universities;
- Student participation in the governance of higher education;
- Public responsibility for higher education;
- The importance of the social dimension of the Bologna Process (Bologna Process 2004: 2).

The understanding and practical pursuit of fundamental values in the EHEA are not unproblematic. In this particular case, it should be noted that there is, of course, a difference between fundamental values and underpinning principles that can explain some of the differences between the two lists above, but this difference cannot explain, for example, the absence of reference to academic freedom or to staff participation in governance in the 2004 BFUG document. In fact, academic freedom is a particularly pressing matter regarding both the present and the future of the EHEA.

The discussions about the future also touch on the question of whether or not the EHEA, and the project behind it, is a success or failure (Matei 2018), whether it has been completed or not, and whether the EHEA is a settled reality or one that is still in movement. Seeing the EHEA as a static area would ignore two essential facts: it continues to develop, and the implementation of policies adopted and defined through successive Ministerial communiqués is imperfect and remains so even a decade after the formal launch (European Commission//EACEA/Eurydice 2018).^4^

As part of the development of the EHEA, its terminology has also evolved. Luckily, what was originally referred to as the “external dimension”, making a clear distinction between “them” and “us”, is now generally referred to as the “global dimension” or “the EHEA in a global setting” (Bologna Process 2007). Nevertheless, we would argue that the Policy Forum, as a tool to promote this external dimension, has not found a form that makes it an attractive platform for cooperation between the EHEA and other regions of the world. A suggested change of name to the Global (rather than “Bologna”) Policy Forum for the 2020 edition is unlikely to change this perception.

Along with the note about the incompleteness of the implementation of EHEA and the related many difficulties and shortcomings, it is also important to note a series of remarkable achievements. There was good reason to celebrate the 20th anniversary of the Bologna Declaration, as was duly done in June 2019, and appropriately at the University of Bologna.^5^ There was much to celebrate; the Bologna Process has changed higher education in Europe in ways those who signed the Bologna Declaration could probably not quite have imagined (as confirmed by at least three of them^6^ who were present at the celebration). As ministers responsible for higher education in all 48 EHEA countries prepare to gather in Rome a year later, in 2020,^7^ the focus will nevertheless be more on the challenges ahead, on the future of the EHEA, than the achievements of the past. These challenges are also the focus of the session on “The future of the EHEA—principles, challenges and ways forward” at the 2020 edition of the Bologna Process Researchers’ Conference, which we had the honor to coordinate. The chapters in this section of the current volume are based on papers presented in this session about “the future”. They offer a variety of research-informed perspectives on the future of the EHEA, trying to make a contribution to this topic that goes beyond either the desolate or the enthusiastic talk about any *fata morgana.*

Based on research, systematic scrutiny and analysis, the papers also discuss possible further developments. In a gesture of engagement and responsibility, they try to identify possible lessons and ways to address continuing and new challenges. There are also implicit or even direct recommendations for possible courses of action that are put forward.

## Changing Contexts, Emerging Issues

The Bologna Declaration and its emphasis on structural reforms to improve completion rates as well as international mobility responded to urgent issues with which most European countries were faced at the turn of the millennium. These were issues that could be addressed through fairly loosely organized cooperation in a policy area in which national authorities are jealous of their prerogatives, as shown for the EU member states, at least, through the Maastricht Treaty (Council of the European Communities/Commission of the European Communities 1992). Education is one of the areas in which the EU does not have exclusive competence and where it was considered preferable to apply the principle of subsidiary contained in the EU Treaty.^8^

Two decades later, European societies have evolved, and new issues have moved to the forefront of the professional, policy and political debates in higher education. Implementation of structural reforms—a major motivation for starting the Bologna Process in the first place—remains important, both because these reforms are essential and because putting them into practice takes longer than originally foreseen. Issues around the main approach to implementation gave rise to what was probably the most heated discussions ever in the BFUG in 2015–2018. In essence, the argument was between those who favored recourse to peer learning alone to promote the EHEA objectives and those who, while accepting the importance of peer learning, wished to see it complemented by more explicit follow-up of EHEA member countries that were far from implementing specific commitments, as shown by the Bologna Implementation Reports. The discussion resulted in a “[s]tructured peer support approach for the implementation of the three Bologna key commitments” (Bologna Process 2018: 5) and the setting up of a Bologna Implementation Coordination Group in the 2018–2020 work program.^9^

More broadly, the contentious debate on implementation reflects divergent views of the character of the EHEA itself. What does it mean that the EHEA is a voluntary cooperation? Is it voluntary to join, but once a country joined, it is required to implement its commitments, or are EHEA members free to consider commitments rather as optional guidelines and policy aspirations (Bergan 2015; Bergan and Deca 2018; Harmsen 2015; Vidjarsdóttir 2018)? Even if the focus on peer support in the 2018–2020 work program was considered a reasonable compromise by most of those engaged in this difficult debate, the underlying different approaches to the EHEA remain and must be expected to color, at least in part, the further debate on the “future of Bologna”. One of the key points of discussion in the section on “the Future of the EHEA” at the 2020 Bologna Process Researchers’ Forum was whether peer support and peer learning are by themselves sufficient measures to ensure that the fundamental values of the EHEA are respected. While we would not claim there was consensus among participants, those who spoke on this issue tended to believe that stronger instruments will be required for the future. At the risk of upsetting a compromise that was reached at great expense of energy and adrenalin in the BFUG, this discussion is likely to continue into the next decade of the EHEA, as it will be at the core of a continuing debate not only about what it means to be an Area but also what it means to be European.

At the same time, in addition to older issues (which one may call the original or foundational “Bologna sins”), new ones emerge that are also linked to the very character and organization of the EHEA. Some of them do, for example, raise questions about whether a relatively loosely organized cooperation between public authorities (remember that the EHEA is considered to be a voluntary inter-governmental initiative) is the right forum for addressing them. The financing of higher education is undoubtedly an important issue in all countries, and countries can learn from each other’s experience (Matei 2012), but it is less clear that developing common guidelines or commitments in the framework of the EHEA would be the best course of action, beyond the affirmation of higher education as a public good and public responsibility (Bologna Process 2001, 2003) and a commitment to “securing the highest possible level of public funding for higher education and drawing on other appropriate sources” (Bologna Process 2010: 1).

Another set of issues is linked to learning and teaching (covered in another section of this volume). Learning and teaching are, of course, one of the prime tasks of higher education, but the main actors are students, faculty, and institutions—not the public authorities. As far as public authorities are concerned, the main question is whether they can help at all, and then how to help develop good practice in learning and teaching in the EHEA through incentives and through reform of the education systems. A particular issue concerns the use of Artificial Intelligence and, more broadly, information technology, which is no longer labeled a “new technology”. Many higher education institutions are of course well advanced both in research in these areas and in using the technologies in learning and teaching. We would also argue that quality education will in the future depend largely on the extent to which programs and institutions have recourse to a variety of pedagogies and modes of delivery: the question is less whether learning and teaching should be online or face to face than how programs and institutions make use of both. Again, the challenge for the EHEA in the immediate future is to define how public authorities can, through a relatively loosely organized European cooperation, best further policies and practice in an area in which students, faculty, and institutions are the main actors.

The social dimension of higher education has been on the “Bologna agenda” since the Prague Ministerial Conference (Bologna Process 2001). Since 2015, the social dimension has been linked more explicitly to the broader societal mission of higher education. In Yerevan, Ministers underlined that

**Making our systems more inclusive** is an essential aim for the EHEA as our populations become more and more diversified, also due to immigration and demographic changes. We undertake to widen participation in higher education and support institutions that provide relevant learning activities in appropriate contexts for different types of learners, including lifelong learning (Bologna Process 2015a: 2; bold in the original).

In Paris, they stated:

We recognise that further effort is required to strengthen the social dimension of higher education. In order to meet our commitment that the student body entering and graduating from European higher education institutions should reflect the diversity of Europe’s populations, we will improve access and completion by under-represented and vulnerable groups (Bologna Process 2018a: 4).

At the same time, the broad agreement that the social dimension of higher education is important has not been matched by agreement on actual policy measures, whether at the European or national level (European Commission//EACEA/Eurydice 2018: 214). In June 2020, Ministers are expected to adopt “European Principles and Guidelines to Strengthen the Social Dimension of Higher Education” (Bologna Process 2020a), which are currently available in draft form and which will be further discussed by the BFUG.

The fundamental values of the EHEA were once taken for granted but have now surfaced as one of the most difficult issues facing the EHEA. The reason is that over the past few years, we have seen increasing violations of these values, as underlined in the Paris Communiqué:

Academic freedom and integrity, institutional autonomy, participation of students and staff in higher education governance, and public responsibility for and of higher education form the backbone of the EHEA. Having seen these fundamental values challenged in recent years in some of our countries, we strongly commit to promoting and protecting them in the entire EHEA through intensified political dialogue and cooperation (Bologna Process 2018a: 1).

The situation of the Central European University, which has been forced by a series of politically motivated actions initiated by a national government to move most of its teaching and learning and research activities from Hungary to Austria, is an emblematic example but there is, alas, no shortage of others, such as legislation in Turkey and Hungary more broadly or the revoking of the license of the European University in St Petersburg in March 2017 by Russian authorities. We mention these examples because they are the ones included in the latest Bologna Implementation Report (European Commission//EACEA/Eurydice 2018: 42), but the list is far from complete. There is even talk about a crisis of academic freedom presently in the EHEA altogether (Matei 2020). It is also worth recalling that implementation of the fundamental values of the EHEA was included in the Belarus Roadmap (Bologna Process 2015b) and that the assessment of the implementation of this part of the Roadmap was critical (Bologna Process 2018b: 15). Aspects of the fundamental values of higher education have also been the topic not only of the work of the Magna Charta Observatory,^10^ but also of other organizations. In June 2019, the Council of Europe and other partners organized a Global Forum in Strasbourg on academic freedom, institutional autonomy, and the future of democracy (Council of Europe 2019; Bergan et al. forthcoming).

As with the social dimension of higher education, however, agreeing that the fundamental values are and should be at the core of the EHEA does not easily translate into agreed policy or performance criteria, as illustrated by the quite perfunctory coverage of these issues in the latest Bologna Implementation Report (European Commission//EACEA/Eurydice 2018: 40–46; see also Jungblut, Maassen and Elken in this volume). The BFUG therefore appointed a Task Force to put forward recommendations for future monitoring of values. The current draft (Bologna Process 20120b) is still under discussion in the BFUG; the intention is to submit a proposal for adoption at the June 2020 Ministerial Conference.

The discussion of fundamental values is challenging not only because they touch on the soul of the EHEA but also because they concern the identity of its member countries and their commitment to this common space of policy dialogue and practice. It may be painful for a minister to recognize that his or her country lags behind in developing its qualifications framework or quality assurance arrangements, and the discussions in the BFUG on implementation and non-implementation between 2015 and 2018 underscored the point. Nevertheless, for a minister to admit that his or her country is deficient in academic freedom, institutional autonomy, or student and staff participation in higher education governance—as judged against clear European standards and common references—is infinitely more difficult, as it amounts to admitting openly that the country is less than democratic. It is perhaps a sign of health that making this admission is difficult, but it is not a sign of societal health that some governments try to make a virtue of being less than democratic, sometimes by attempting to redefine democracy by combining it with alien concepts like “illiberal”.

## Where Is the EHEA Heading?

This question, which has been asked almost since the Bologna Process was launched, is approached from three quite different angles in the present volume.

Writing from the multiple perspective of a long-time professor of history at an Italian university and a leading actor in international projects like TUNING as well as her current experience as Vice Chair of the BFUG, Ann Katherine Isaacs looks at the major challenges with which the EHEA will be faced in the next decade. One of them is that while discussions of new priorities have often faced on which specific issues should be addressed, the EHEA now needs to develop (finally!) a convincing vision. Focusing on the ongoing discussion within the BFUG, the author considers how this group—and by extension the ministers meeting in Rome in June 2020—could develop such a vision for the EHEA for the next decade. She also explores whether this vision could be furthered by reference to a European higher education community or system.

Approaching the success and the challenges of the EHEA from a political science perspective, Jens Jungblut, Peter Maassen, and Mari Elken argue that there is good reason to celebrate the first two decades of the EHEA and underline that it has played an important role in reforming the higher education structures in Europe. At the same time, it faces serious challenges, not least as concerns identification with and respect for its fundamental values. In this area, the situation is even more challenging now than when the Bologna Process was launched or the EHEA formally established. A shift in focus from the structural and technical progress made to underlying political tensions and conflicts would coincide with a declining political interest in the EHEA in most member states, in spite of the fact that the 2018 Ministerial Conference in Paris had stronger political representation than the conferences immediately preceding it.

Ligia Deca and Robert Harmsen also use a political science concept—soft governance—in their analysis of the EHEA. They see the EHEA as a relatively successful example of soft governance. Despite being successful in many ways, however, the EHEA is faced with challenges that touch on its very direction and purpose, as exemplified by the often bitter debate on implementation/ non-implementation in the 2015–2018 work period. The authors also explore the role of the EHEA as a policy forum and a community of values. The discussion about the nature of the EHEA also links to the broader debate about “Europe”—often meaning the EU—and cannot remain untouched by current trends toward Euroscepticism. This trend is most explicitly exemplified by Brexit but is found in many EU member States.

## Specific Challenges Toward 2030

A subset of articles considers specific aspects of the development of the EHEA, ranging from autonomy and accountability through the organization of studies and quality assurance to the challenges of establishing a more independent Secretariat not linked to the hosts of the upcoming Ministerial Conference.

Veronika Kupriyanova, Enora Bennetot Pruvot and Thomas Estermann explore the relationship between autonomy, efficiency and accountability. Drawing on the EUA’s work on institutional autonomy and the University Autonomy Scorecard as well as the higher education efficiency framework developed by the EUA, the authors explore the impact of regulatory frameworks on efficiency in institutional management, how autonomy can be used to enhance efficiency and effectiveness, and how efficiency can support autonomy. They consider all issues in relation to the various dimensions of autonomy developed by the EUA Scorecard: organizational, financial, staffing and academic autonomy. They suggest the capacity of institutions to manage funds internally, select and promote their staff, and design their academic offer to match the needs as institutions identify them are key success factors.

In the context of the discussion about present and future challenges in the EHEA and common values in higher education, Liviu Matei explores what he calls the “crisis of academic freedom” in the EHEA. His paper also looks at actual and potential efforts to overcome this crisis, in its two dimensions: intellectual (academic freedom has been severely neglected in EHEA intellectual and policy debates, there is no common conceptual reference for academic freedom in the EHEA) and empiric (academic freedom is challenged, threatened or directly under attack in almost all regions of the EHEA). Designing a way out of the crisis requires a coordinated effort of charting a new course for academic freedom. A comparative analysis of who is doing this, who is charting the shape or course for academic freedom in Europe and the United States, reveals surprising differences. The most striking of all is the complete absence of higher education institutions themselves in Europe from these discussions and efforts aiming at charting a course for academic freedom.

Tim Birtwistle and Robert Wagenaar explore the impact on higher education learning of the quite extensive and rapid changes in society and the labor market we are currently witnessing. To meet these challenges, higher education institutions—as well as public authorities as custodians of education systems—should reassess the way generic and subject specific competences are combined and balanced. Even more, however, systems and institutions will need to broaden opportunities for lifelong learning by enabling learners to design their own learning pathways based on three key components: (1) a core focused on a particular field of studies (thematic or disciplinary); (2) a fully integrated set of transferable skills; and (3) a large set of learning units of various sizes covering a flexible curriculum. They suggest developing a broad offer of micro-credits should be an important part of this effort.

Sjur Bergan and Irina Geantă discuss the feasibility of setting up a permanent Bologna Secretariat, in the light of the broader challenges to the EHEA over the next decade. While this issue has been considered by the BFUG as well as by a previous Bologna Process Researchers’ Conference (Bergan 2015), this discussion has so far not moved much beyond listing potential obstacles. The authors explore challenges related to the tasks of a permanent Secretariat as well as a set of issues related to its status, location, financing, and staff. They consider relations to the authorities of both the country hosting the Secretariat and those hosting successive Ministerial Conferences. Without arguing that a permanent Secretariat is a necessary condition for the EHEA to develop further, the authors identify a set of conditions they argue must and could be fulfilled if the BFUG—and more broadly the EHEA—is to be served by a more stable Secretariat than one provided by and linked to the hosts of the forthcoming Ministerial conference.

## Conclusion

An emphasis on defining an overarching vision for the EHEA as we look toward its third decade should be welcomed. This vision should include, among other things, a more precise and careful conceptual and policy articulation of the fundamental values of higher education in the context of the EHEA and a more clear, flexible but workable governance structure. The EHEA has largely been successful in devising and implementing structural reforms, even if the continuing bitter debate on implementation shows that putting policies into practice has been difficult, and success has been less than complete.

However, both the reforms carried out so far and successive discussions of new priorities have largely been presented as a set of individual measures, perhaps with the Working Groups on “new goals” in the 2015–2018 period (Bologna Process 2018c) as a particularly poignant example of measures that each had their merits, but the rationale for which was not argued in terms of how they would develop the EHEA as such.

Our contention is that while the EHEA has carried out successful reforms in the past and demonstrates potential for the next decade, it has lacked the will or the ability to couch these in terms of an overall rationale or vision. Structural reforms all imply technical challenges, and these have to a considerable extent been met. But structural reforms are also undertaken for a broader purpose, or at least they should be. In the case of structural reforms, part of the rationale has in fact been articulated: reducing drop-out rates and providing both students and employers with competences and qualifications at different levels. Except for doctoral qualifications, which are at the top of the qualifications framework and therefore do not lead to further formal qualifications even if they often lead to a lifetime of learning and research, every qualification in the higher education system has two objectives: qualifying for further study, i.e. access to a study program at a higher level, and qualifying for appropriate and meaningful employment. As important as these purposes are, however, they have not been articulated in relation to a higher purpose: how education contributes to developing the kind of society in which we would want to live.

Until the Ministerial Conference in 2020 and the adoption of the Rome Communiqué, the jury will perhaps be out on whether the next decade of the EHEA will be based on a coherent vision. In closing this introduction to the consideration of the “future of Bologna” at the 2020 edition of the Bologna Process Researchers’ Conference, we nevertheless venture to offer our own view on what the EHEA of the next decade should strive for.

We cannot imagine an EHEA that would truly serve European societies at large, its academic community of scholars and students, and its democratic traditions unless it offers students, staff, and graduates an opportunity to move freely throughout all of the EHEA for purposes of work or study.

Committing to this seemingly simple vision has much broader implications than what may appear at first sight. We cannot move freely unless the full value of our qualifications is recognized, so that we do not have to leave part of their real value behind at the “border” between systems because of less than fair recognition practices, incompatible qualifications frameworks, or lacking quality assurance mechanisms—so the structures of our education systems will still be essential. We cannot move freely if the ways in which we learn and teach do not encourage us to reflect critically or if they ignore technological developments and their opportunities as well as their pitfalls. We cannot move freely if our education systems and institutions do not enable all of us to develop our potential and aspirations to the full, either because of barriers to access or because of barriers to successful completion.

Most importantly, we cannot move freely if the political conditions of the EHEA block us. Democracy and education quality both require academic freedom and institutional autonomy. Higher education must help develop a culture of ethics, transparency and integrity, and it must draw on the contributions and creativity of students and staff by involving hem in higher education governance.

As we write these lines, the importance of higher education and research has been illustrated in a quite dramatic way. The transmittable disease that has become known as the COVID-19 pandemic shows in at least four ways why higher education and research are essential. Firstly, we need to improve our knowledge and understanding of this particular virus through research. At the time of writing, there are simply too many things we do not know about this virus to take fully effective measures, even if research seems to be progressing relatively fast, building, of course on basic research that has been developed over generations. Secondly, we need to disseminate the knowledge and understanding we *do* have about the virus, and also an understanding of the limits of that knowledge, among non-specialists. Higher education must play a key role here. Thirdly, public authorities must develop policies to meet the threat posed by the virus in reasonable and efficient ways, but without overreacting. Again, this cannot be done without the contribution of higher education and research. And, fourthly, we must meet the kind of populist reactions that seem to believe the disease is carried through a specific nationality or passport with arguments that are easy to understand but nevertheless based on facts that can be complex and that withstand the test of democratic debate.

The example of the COVID-19 virus provides an urgent example not only of why higher education and research are important, but also of why a European Higher Education Area built on structures and values, teaching and learning, excellence and inclusion is essential to our future.

A European Higher Education Area that were not built on and did not help develop a culture of democracy, and hence were not respectful of academic freedom, institutional autonomy, ethics and transparency, and student and staff participation in higher education governance, would not provide quality education and would not help build the kind of society in which we would like to live ourselves or that we would want to leave to our children or grandchildren.

Our challenge, then, is to build on the first two decades of the EHEA to make sure that when it reaches the age of 30, it will be an area of coherent higher education policy and practice that makes Europe not only competitive but inspiring, an area others will not only want to compete against but to be inspired by and emulate.

## Footnotes

1 The Bologna Process, launched with the Bologna Declaration of 1999, is a voluntary intergovernmental process in higher education based on jointly agreed principles, objectives and standards. Currently, there are 48 European states implementing the Bologna Process, which constitute the European Higher Education Area (EHEA); a 49th country (San Marino) may accede at the June 2020 Ministerial conference. The EHEA, as the common European space for higher education, is considered a result of the Bologna Process and it has formally been in existence since 2010.

2 http://www.ehea.info/pid34364/bologna-policy-forum.html, accessed February 27, 2020.

3 http://www.ehea.info/page-the-bologna-follow-up-group, accessed February 27, 2020.

4 The 2018 Implementation Report is the latest one available at the time of writing. A new Implementation Report will be published in time for the 2020 Ministerial Conference, but it will have a slightly different scope than previous reports in that it will focus on some longer-term trends in the development of the EHEA.

5 http://bolognaprocess2019.it/, accessed February 27, 2020.

6 Agneta Bladh (Sweden), Tatjana Koķe (Latvia) and Pavel Zgaga (Slovenia).

7 The Ministerial conference has now been postponed from June to November 2020 because of the COVID pandemic.

8 https://www.europarl.europa.eu/factsheets/en/sheet/7/the-principle-of-subsidiarity, accessed February 27, 2020.

9 http://www.ehea.info/page-Bologna-Implementation-Coordination-Group, accessed February 27, 2020.

10 http://www.magna-charta.org/, accessed February 27, 2020.
